# Elucidating the 3D structures of Al(iii)–Aβ complexes: a template free strategy based on the pre-organization hypothesis†
†Electronic supplementary information (ESI) available: Detailed computational details, geometry data of all 3D Al(iii)–Aβ models, supplementary figure and table. See DOI: 10.1039/c7sc01296a



**DOI:** 10.1039/c7sc01296a

**Published:** 2017-05-09

**Authors:** Jon I. Mujika, Jaime Rodríguez-Guerra Pedregal, Xabier Lopez, Jesus M. Ugalde, Luis Rodríguez-Santiago, Mariona Sodupe, Jean-Didier Maréchal

**Affiliations:** a Kimika Fakultatea , Euskal Herriko Unibertsitatea (UPV/EHU) and Donostia International Physics Center (DIPC) , 20080 Donostia , Euskadi , Spain . Email: joni.mujika@ehu.eus; b Departament de Química , Universitat Autònoma de Barcelona , Bellaterra 08193 , Spain . Email: JeanDidier.Marechal@uab.cat

## Abstract

Senile plaques are extracellular deposits found in patients with Alzheimer’s Disease (AD) and are mainly formed by insoluble fibrils of β-amyloid (Aβ) peptides. The mechanistic details about how AD develops are not fully understood yet, but metals such as Cu, Zn, or Fe are proposed to have a non-innocent role. Many studies have also linked the non biological metal aluminum with AD, a species whose concentration in the environment and food has been constantly increasing since the industrial revolution. Gaining a molecular picture of how Al(iii) interacts with an Aβ peptide is of fundamental interest to improve understanding of the many variables in the evolution of AD. So far, no consensus has been reached on how this metal interacts with Aβ, partially due to the experimental complexity of detecting and quantifying the resulting Al(iii)–Aβ complexes. Computational chemistry arises as a powerful alternative to investigate how Al(iii) can interact with Aβ peptides, as suitable strategies could shed light on the metal–peptide description at the molecular level. However, the absence of any reliable template that could be used for the modeling of the metallopeptide structure makes computational insight extremely difficult. Here, we present a novel strategy to generate accurate 3D models of the Al(iii)–Aβ complexes, which still circumvents first principles simulations of metal binding to peptides of Aβ. The key to this approach lies in the identification of experimental structures of the isolated peptide that are favourably pre-organized for the binding of a given metal in configurations of the first coordination sphere that were previously identified as the most stable with amino acid models. This approach solves the problem of the absence of clear structural templates for novel metallopeptide constructs. The posterior refinement of the structures *via* QM/MM and MD calculations allows us to provide, for the first time, physically sound models for Al(iii)–Aβ complexes with a 1 : 1 stoichiometry, where up to three carboxylic groups are involved in the metal binding, with a clear preference towards Glu3, Asp7, and Glu11.

## Abstract 2

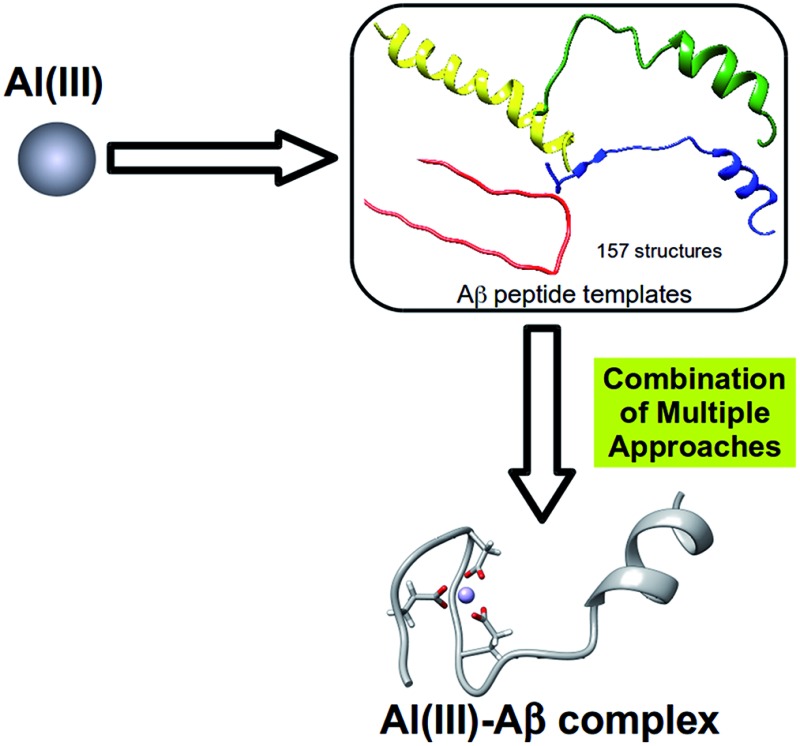
We present a novel strategy to generate accurate 3D models of Al(iii)–Aβ complexes, which circumvents first principles simulations of metal binding to peptides of Aβ.

## Introduction

The quest for cures of neurodegenerative diseases has become a vital field of research in our modern societies and many directions are considered to fight against them, from genetic to pharmacological approaches. Whatever the strategy, decoding key cellular and molecular mechanisms of sickness represents a fundamental objective in this field. Amongst the molecular aspects shared by most neurodegenerative diseases is the formation of insoluble peptide aggregates. These species are formed through the association of soluble protein fragments known as amyloids. About twenty proteins are already known to form such amyloids as Aβ in Alzheimer’s disease (AD) or α-synuclein in Parkinson’s disease (PD).

In AD, two main types of deposits are found: senile plaques and neurofibrillary tangles. The former contains insoluble filaments made of β-amyloid fragments and the latter consists of hyperphosphorylated tau proteins. The γ-secretase enzyme acts on the Amyloid Precursor Protein (APP) membrane protein to produce the Aβ_1–40_ and Aβ_1–42_ fragments that are found in senile plaques. Despite the fact that the formation of these peptides is a normal process in healthy people, several factors may prompt an imbalance in their concentration and so favoring and accelerating the aggregation in the unhealthy ones. The aggregation is a complex process in which species such as soluble oligomers, paranuclei, protofibrils, and finally insoluble filaments are produced.^1^

As it has been recently discussed in the review by Kepp,^2^ three main hypotheses arise to explain the main factors of AD development: the amyloid cascade hypothesis, the oxidative stress hypothesis, and the metal ion hypothesis.^3–5^ The metal ion hypothesis claims that metals naturally involved in biological systems, like Fe, Cu, and Zn, have a key role in AD and a large body of evidences indicates that they participate in different stages of the formation of amyloids and their aggregated forms.^6–8^ As knowledge about AD increases, other metals coming from human activities are now the usual suspects in the evolution of the disease. Among them, Al(iii) has become the main focus of attention. In spite of its absence from our evolutionary process, human intervention (water treatment, acid rain, drugs, *etc.*) has increased its bioavailability and its exposure into organisms in which it may interact at cellular and molecular levels.^9^

How aluminum is involved in AD is still unclear although it has been associated with different functions.^10–13^ Aluminum has been detected in senile plaques extracted from the brains of patients with AD^14^ and several *in vitro* experiments showed that Al(iii) promotes aggregation more efficiently than other metals.^15–19^ The analysis of Aβ aggregates using transmission electron microscopy reveals that Al(iii) favors the formation of smaller oligomers than the ones made by Cu, Zn, or Fe.^19^ Moreover, it was shown that the Aβ–Al complex alters the cell morphology and increases the membrane fluidity.^19^ These authors indicated that the small size of the Al(iii)–Aβ oligomers combined with their high hydrophobicity may facilitate the crossing of the complex across the cell membrane and, once there, the Aβ fragments may lead to pathological effects. In addition, it has been determined that the amount of Aβ peptides crossing the blood–brain barrier increases by 60% when they are bound to Al(iii).^20^ Nevertheless, most of the studies on the Al(iii)–Aβ complex report whether the Aβ peptide interacts with Al(iii),^21–24^ or analyzed the physiological morphology adopted by the aggregates.^17,18,25–27^ However, to the best of our knowledge there is no investigation on the particular mode of coordination of Al(iii) with Aβ peptides at the molecular level. This lack of data is due in part to the inherent difficulties of characterizing the Al(iii) coordination shell. In this vein, computational chemistry could become an interesting ally in shedding light on the interaction mode between Al(iii) and Aβ peptides.

The prediction of metal binding processes to biomolecules is one of the most challenging exercises that computation could answer. The quest is even harder when dealing with small peptides, because the variability of the first coordination sphere of the metal upon binding is complicated by the intrinsic flexibility of the polymer. Therefore, numerous intermediates could occur along the simulation of metal – binding complexation and first principles simulations are therefore extremely complex. Biased computations appear to be necessary but are also challenging since suitable approximations are needed to drive the modeling. One possible procedure to escape these limitations consists of using molecular mechanics force field-based computational methods to reach physically sound starting point geometries of the metallopeptide, and then refine the best possible candidates with more accurate calculations like hybrid quantum mechanics/molecular mechanics approaches. The key element of this kind of procedure resides in accounting for the appropriate amount of starting point structural information to narrow the conformational space to explore.

This strategy has been our focus in several recent studies like the prediction of different copper binding Aβ complexes where NMR structures of Zn(ii) bound to Aβ peptides were used as templates for an initial homology modeling exercise.^28^ However, it is easy to foresee that the same protocol is not valid for building Al(iii) metallopeptides. According to the HSAB theory, Zn(ii) and Cu(ii) are acids of intermediate strength and Al(iii) is a hard acid. Consequently, the chemistry of the latter differs from the chemistry of the other two metal ions naturally occurring in biological systems; Al(iii) shows a preference for negatively charged oxygen containing groups, such as phosphates and carboxylates,^29^ while Zn(ii) and Cu(ii) tend to interact with N containing groups, as the well established coordination modes of these two cations to the Aβ peptide demonstrate.^7,30^ As a consequence, the unique experimental structure of the Zn–Aβ complex available in the Protein Data Bank^31^ does not represent a possible starting point for building a 1 : 1 stoichiometric fold of aluminum interacting with β-amyloid. This is a major missing piece of information when aiming to decode the structures of Al(iii)–Aβ.

A possible way forward lies in identifying pre-organized structures of the metal free peptide that could host the ion in its best possible coordination environments, and from there generate final metallopeptide models. The basic idea of this approach stands on the possibility of any receptor naturally exploring, at room temperature, microstates that are close to the metal complexed geometries. This concept is recurrent in many molecular fields nowadays from protein–ligand interactions (*i.e.* in the conformational selection hypothesis),^32,33^ *de novo* enzyme design,^34–36^ metal binding to proteins,^37^ cyclic peptides,^38^ supramolecular chemistry,^39^ and inorganic chemistry especially when dealing with multidentate ligands.^40,41^ Although the exact contributions of pre-organization and induced effects in the binding processes are not yet clear – likely depending on the system under investigation – the former offers a possible solution to the absence of a structural template for the Al(iii)–Aβ system. Indeed, plenty of structures of the sole Aβ peptide presenting alternative conformations are available in the Protein Data Bank^42^ and suitable algorithms could help in identifying those that satisfy the geometric conditions for the best arrangements of the first coordination sphere of aluminum.

Here, we perform a multi-level computational study that ranges from quantum mechanical cluster models to full size system QM/MM calculations and is based on the host–guest pre-organization hypothesis. After decoding the nature of the most stable first coordination sphere of the metal with a prototypical ligand in biological media (*i.e.* amino acids and water) *via* QM calculations, the Protein Data Bank is screened to find geometries of the peptide that could satisfy the requirement of the best first coordination sphere environments. Then a series of plausible candidates are selected and finally refined with QM/MM approaches, hence providing a certain amount of induced fit effect. The results of our study provide key insights regarding the formation of Al(iii)–Aβ complexes, suggest physically sound atomistic scale models of these systems, and add to the still limited panel of molecular tools available for metallopeptide studies with a novel procedure based on an updated molecular hypothesis.

## Computational details

**Fig. 1:**
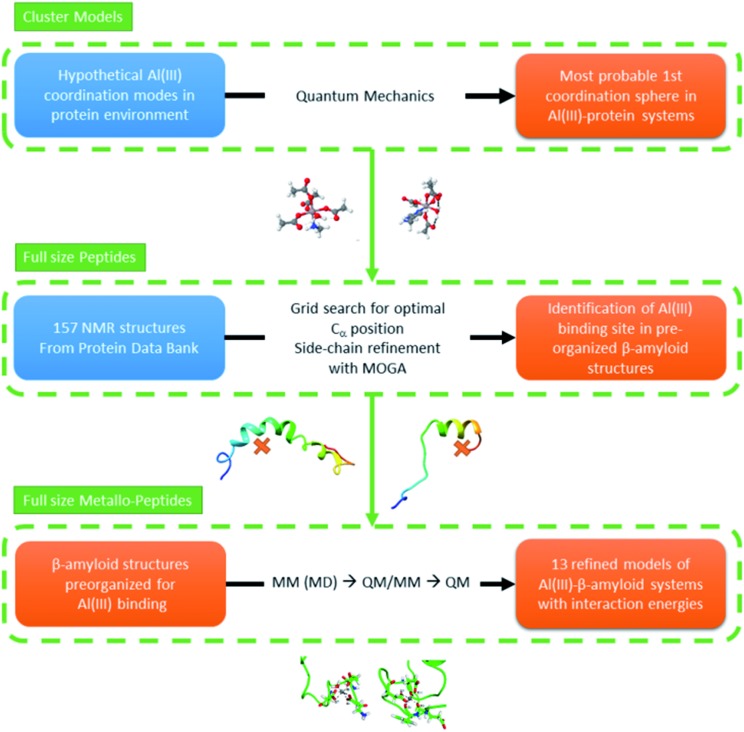
Schematic illustration of the strategy followed throughout the present work.

In this section we present our strategy to reach reliable models of Al(iii)–Aβ, and this consists of a multi-level protocol where accurate computational chemistry approaches are combined with structural statistic searches. Full technical details are available in the ESI.†The overall methodology (summarized in Fig. 1) can be divided into three successive steps.

### 1. Characterization of the most favorable first coordination spheres of aluminum with amino acid motifs

Only scarce information is available on how Al(iii) interacts with biological motifs. Nonetheless, this is a fundamental piece of knowledge when aiming at generating trustworthy models of the interaction of this ion with Aβ peptides. Therefore, a series of first coordination sphere hypotheses were explored *via* accurate quantum mechanics methods using a cluster model approach. Numerous models that account for the side chain, main chain, and water coordination with Al(iii) throughout nitrogen and oxygen coordination were optimized and their energies compared. Only the few lowest energy systems where considered as possible environments for an *in vivo* condition for the full length Al(iii)–Aβ system.

### 2. Selection of full length amyloid structures compatible with optimum aluminum first coordination spheres

Hundreds of NMR and X-ray structures of peptides, including Aβ forms, can be found in the Protein Data Bank.^42^ However, experimental structures of metal-bound peptides are scarce and to the best of our knowledge only a few Zn(ii)–Aβ systems can be found in this database.^43–45^ As direct metal mediated folding of a peptide is not yet viable by computational means (*i.e.* MD) we decided to look for amyloid structures that display convenient scaffolds to host an octahedral aluminum. By doing this we aim to identify, within the ensemble of experimental structures available in the PDB, those structures that present the relevant pre-organization of the peptide to satisfy the Al(iii) environment of lower energy, as detected in step one. This was performed by: (1) selecting Aβ peptides from the experimental PDB bank database,^42^ (2) filtering of the structures *via* a grid protocol to detect suitable arrangements of backbone atoms for coordinating an Al(iii), keeping the coordination mode predicted by cluster model calculations, and (3) generating refined 3D candidates for the Al(iii)–Aβ structure. For the last point, we set up and applied an in-house made genetic algorithm to identify the best position of the metal and the correct orientation of the side chains to generate an adequate Al(iii)–Aβ model.

### 3. Refinement of the Al(iii)–Aβ structures and calculation of interaction energies

Once a set of Al(iii)–Aβ models were built, their structures were equilibrated using molecular dynamics simulations. Three structures from each MD simulation were subsequently minimized using a QM/MM protocol. Afterwards, the interaction energy between Al(iii) and the Aβ peptide of each complex structure was determined using pure QM calculations. Since in all of the complexes the 1–11 segment of the peptide accommodates the cation binding site, the 1–16 segment of the Aβ peptide was taken to calculate the interaction energy according to reaction (1), where Aβ_1–16_ corresponds to the 1–16 fragment of the apoform of the Aβ peptide, [Al·Aβ_1–16_·(H_2_O)_6–*m*_] is the complex formed by the fragment with Al(iii), and *m* refers to the number of water molecules displaced by the Aβ peptide from [Al·(H_2_O)_6_]. Notice that the structure of the apoform was taken from the complex, and therefore reaction (1) accounts for the interaction energy between the Aβ peptide and Al(iii) in the [Al·Aβ_1–16_·(H_2_O)_6–*m*_] complex structure.

1 [Al·(H_2_O)_6_]^3+^ + [Aβ_1–16_]^1–^ ↔ [Al·Aβ_1–16_·(H_2_O)_6–*m*_]^2+^ + *m*·H_2_O

## Results

### 1. Small QM cluster models

**Fig. 2:**
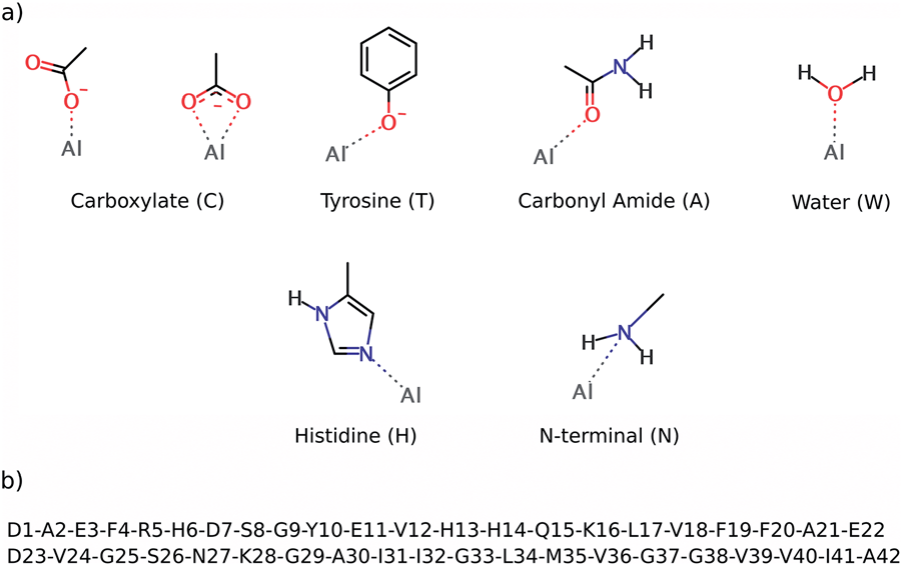
The top image (a) shows the ligands employed to build alternative QM cluster models for the Al(iii) coordination first shell. The below image (b) shows the sequence of the Aβ_1–42_ peptide.

**Fig. 3:**
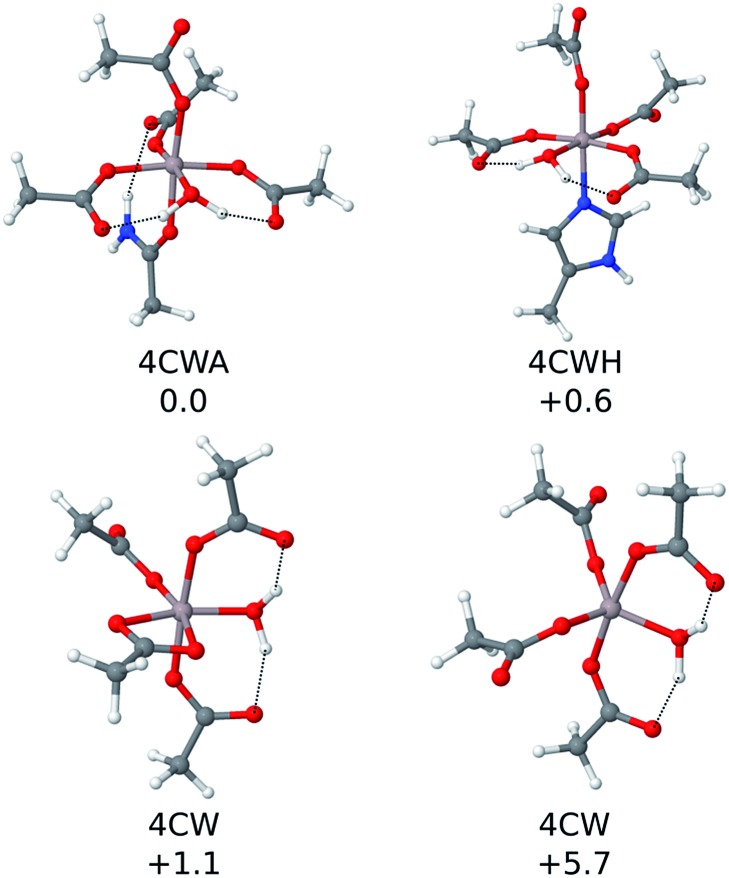
The four most stable QM cluster model structures that were characterized. Their relative ΔΔ*H* ^298^ values (in kcal mol^–1^) are included.

The preferential coordination mode of Al(iii) was investigated by constructing a series of cluster systems where Al(iii) interacts with alternative arrangements of the amino acids of the first coordination sphere. Due to its hard Lewis character, Al(iii) markedly shows a preference towards ligands that donate electrons,^46^ and in particular towards O-containing amino acids, especially carboxylic acids.^47^ Thus, the following building blocks (summarized in Fig. 2) were combined to build the model systems: (a) acetate (C) to mimic aspartic or glutamic acid (both mono- and bidentated to the cation), (b) phenol for tyrosine (T), (c) acetamide for a peptide bond carbonyl amide (A), (d) water (W), (e) 4(5)-methylimidazole for histidine (H), and (f) methylamine for the N-terminal (N). In addition, due to the flexible coordination mode of Al(iii),^48^ the 4, 5 and 6 coordination numbers were considered. In order to reduce the number of possibilities, at least two non-water ligands were included in the model, with at least two carboxylic groups. The stability of each complex was determined by calculating the reaction energy of reaction (2), where *m* refers to the number of ligands which are not ionized (Lig^*n*i^) upon their complexation to Al(iii), while *n* refers to the number of ligands that are considered neutral in solution and unprotonated in the complex (Lig^i^, see below); *p* and *q* refer to the total charge of Lig^*n*i^ and Lig^i^, respectively. The electronic energy (Δ*E*) and enthalpy (Δ*H* ^298^) values of the most stable structures characterized are presented in Table 1 and the four most stable structures are shown in Fig. 3.

2 [Al·(H_2_O)_6_]^3+^(aq,1M) + *m*·[Lig^*n*i^]^*p*^(aq,1M) + *n*·[Lig^i^]^*q*^(aq,1M) ↔ [Al·Lig_*m*+*n*_ (H_2_O)_6–*m*–*n*_]^3+*p*+*q*^(aq,1M) + *m*·H_2_O(aq,1M) + *n*·H_3_O^+^(aq,1M)

**Table 1:** Interaction energies (Δ*E*) and enthalpies (Δ*H* ^298^) (in kcal mol^–1^) between Al(iii) and its coordination sphere computed on DFT/PCM cluster models and evaluated according to eqn (2). The interaction modes of the carboxylic groups are specified as M (monodentated) or B (bidentated). The number of ligands forming each model and the total charge of the system are also specified

Model	Carb.	#Ligand	Charge	Δ*E*	Δ*H* ^298^
4CWA	4M	6	–1	–124.8	–126.1
4CWH	4M	6	–1	–124.1	–125.5
4CW	3M1B	5	–1	–120.7	–125.0
4CW	4M	5	–1	–117.1	–120.4
4CH	3M1B	5	–1	–114.0	–117.8
4C2A	4M	6	–1	–115.0	–117.4
3CWHA	3M	6	0	–115.7	–116.9
4C2H	4M	6	–1	–114.8	–116.5
4CA	4M	5	–1	–112.3	–115.6
4CA	3M1B	5	–1	–112.0	–115.1
3C2WH	3M	6	0	–112.8	–113.7
3C2HA	3M	6	0	–112.1	–113.6
4C	4M	4	–1	–108.6	–113.4
4C	2M2B	4	–1	–108.6	–113.3

The reactants of reaction (2) were considered to be infinitely separated, so the number of ligands enclosed in the complex has a direct consequence for its entropy value, which led to overestimation of the entropic effects. This is especially true in a biological environment, where the reactants are not infinitely separated. Moreover, the calculations on cluster models do not account for the entropic effects ascribed to the conformational change associated with the peptide. So, due to all these factors, the enthalpy values (Δ*H* ^298^) were used to sort out the stability of all of the cluster models characterized. The results confirm that Al(iii) shows a clear preference for carboxylic groups, as four carboxylic groups are present in most of the structures shown in Table 1. Note that tyrosine is the other negatively charged ligand, but its coordination requires being deprotonated first with a subsequent deprotonation energy penalty (Δ*G* ^deprot^ defined in the ESI†), so its coordination to Al(iii) is energetically less favorable than the coordination of a carboxylic group.

The first coordination shell of the four most stable structures (illustrated in Fig. 3), apart from the aforementioned four carboxylic groups, includes one water molecule. This molecule provides extra stability, as it can form strong hydrogen bond interactions with the two carboxylic groups monodentatedly bound to Al(iii). In particular, the most stable structure (4CWA) presents a binding site with four monodentated carboxylic groups, a water molecule and a carbonyl oxygen atom completing the coordination shell of the metal. A similar coordination mode is found in 4CWH, the second most stable structure, where a histidine replaces the carbonyl group. The same 4CW motif is found in the next two structures, and they differ in the coordination mode of one of the carboxylic groups; the bidentated one is about 5 kcal mol^–1^ more stable than the monodentated one. In both structures, the remaining three carboxylic groups interact monodentatedly.

Interestingly, the results point to a penta- or hexacoordinated Al(iii) ion. The Δ*H* ^298^ values of the structures with only 4C groups in the coordination sphere (for instance, 4M and 2M2B structures, the latest two entries of Table 1) are *ca.* –113 kcal mol^–1^, significantly less stable than the structures where the metal coordination shell is completed with additional ligands. Thus, the addition of more ligands to the four carboxylic groups provides an additional stabilization to the system. The carbonyl group appears very often as one of the ligands completing the metal coordination shell. Histidine is also found in some structures.

In summary, the cluster models indicate that the most stable binding site of Al(iii) should provide either three or four carboxylates. The nature of the remaining ligands has a smaller effect, with a water molecule, a carbonyl group, or a histidine being the best candidate. However, it must be pointed out that even though the characterization of the metal binding sites using cluster models provides useful information about the preferred binding site of Al(iii) to an Aβ peptide, these types of calculations do not take into account some key effects, such as the influence of the residues located beyond the first coordination shell, the peptide’s conformation, or the entropic effects. The full Al(iii)–Aβ structure is required to account for these effects (*vide supra*).

### 2. Full size 3D models of the Al(iii)–Aβ complex

#### Building of reliable structures

The DFT/PCM calculations on cluster models shown in the preceding subsection provide us with the preferential coordination modes of Al(iii) in a protein-like environment. However, the effects of the entire Aβ peptide are not included in these models, even though they could drastically modulate the relative order of energy between the different coordination modes and tune the *in silico* predicted best metal–peptide complexes. Thus, the next step along our multi-level strategy consists of building realistic 3D Al(iii)–Aβ amyloid candidates.

Solved structures of Aβ peptides in solution were extracted from the Protein Data Bank (PDB)^42^ and were checked to see to what degree they could adapt to Al(iii). A total of 157 conformations obtained from 13 different NMR samples was considered (summarized in Table S1†). In these structures the Aβ peptide presents a large variety of conformations, thus providing a larger number of templates with which to build a reliable initial structure for the Al(iii)–Aβ complex. Next, a grid search was carried out on all of these structures of the Aβ peptide with the aim of finding a conformation(s) that can be taken as pre-organized enough to be coordinated by Al(iii). The grid exploration identified 194 sites in which Al(iii) could have a viable environment with at least three different amino acid side-chains located consistently with the cluster model results. Note that the C_α_ atoms of the residues were taken as references to give more flexibility to the system (see Computational details in the ESI†). Interestingly, only O-containing amino acids (or water molecules) are found in the vicinity of each of the grid points (potential metal sites) and no nitrogen atoms from the backbone or side chains were identified.

After having reduced the group of possible geometries viable for aluminum binding, a Multi-Objective Genetic Algorithm (MOGA) was applied. As usual with MOGA techniques a high number of potential solutions were thrown (over 37 000 results). Those solutions were filtered in such a way that the maximum clashes do not overcome 30 nm^3^; at least three nitrogen or oxygen atoms would remain in the 2.5 Å from the grid point. Using this criterion the final solutions were reduced to 13, which were then analyzed. Surprisingly, while our algorithm gives the possibility of nitrogenated groups interacting with the metal, none of the 13 solutions present Al(iii)–nitrogen bonds, and aluminium interacts only with oxygen groups. The oxygen atoms can be either part of the Asp or Glu side chains, although they sometimes belong to the backbone. From this initial part of the modeling of the entire metallopeptide it became clear that the Protein Data Bank already contains geometries of the peptides that are sufficiently pre-organized to satisfy the geometric and coordination requirements of octahedral Al(iii) with either aspartates or glutamates coordinated to it.

#### Final models and interaction energies

**Fig. 4:**
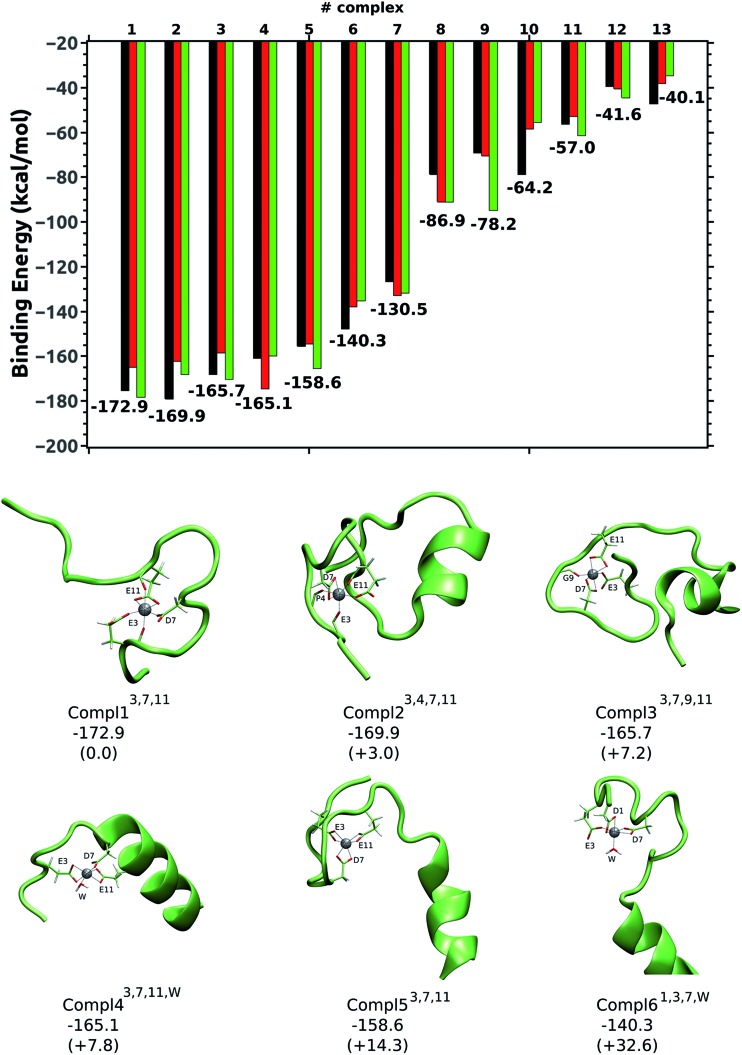
Top image: the interaction energies computed on the three structures optimized for each of the 13 Al(iii)–Aβ complexes. The average interaction energies (in kcal mol^–1^) computed on the three structures are also shown. Lower image: the six most stable Al(iii)–Aβ structures characterized. The interaction energies between Al(iii) and Aβ peptide and their relative differences (in parentheses) are also shown.

The 13 structures chosen from the MOGA search were first solvated with explicit water molecules and then equilibrated using a 10 ns long molecular dynamics simulation. Once equilibrated, three geometries from the MD simulations (two intermediate structures and the last structure of the simulations) were fully optimized *via* a hybrid QM/MM scheme. Finally, the interaction energies between Al(iii) and the Aβ peptide were estimated by performing single-point calculations of the three QM/MM optimized structures, and their average value was computed (more details in ESI†). The computed interaction energies along with the most stable complexes are shown in Fig. 4. For all of the structures characterized, the interaction energies computed on them, along with the distances between Al(iii) and the ligands located in the cation first coordination shell, are listed in Tables S2, S3, and S4†. Note that the structures are labelled as Compl*X*, where *X* indicates their positions in the stability, that is, Compl1 is the most stable one, Compl2 the second most stable, *etc.* The superscripts refer to the residues located in the Al(iii) first coordination shell of the corresponding complex.

Al(iii) presents different coordination modes in the 13 structures, which provides a good scenario to infer the preferential binding mode of the cation. However, all of these structures of the Al(iii)·Aβ complex share a similar pattern for the metal; in all of them Al(iii) appears hexacoordinated (except in Compl9^7,11,*W*^) and interacts only with O-ligands. Moreover, in all of the structures only four of the six carboxylic groups found in the Aβ_1–42_ sequence (shown in Fig. 2) interact with Al(iii), namely Asp1, Glu3, Asp7, and Glu11. Thus, the other two carboxylic groups, Glu22 and Asp23, are predicted not to interact with the cation.

The five most stable structures shown in Fig. 4 present a remarkably more stable Δ*E*_aq_ value than the remaining structures. The energy difference (ΔΔ*E*_aq_) between these five structures is less than 15 kcal mol^–1^, while the ΔΔ*E*_aq_ values of the next two structures are 32.6 kcal mol^–1^ (Compl6^1,3,7,*W*^) and 42.4 kcal mol^–1^ (not shown). Finally, the ΔΔ*E*_aq_ values of the remaining complexes are above 85 kcal mol^–1^ (see ESI†). Therefore, the five most stable complexes are clearly the most representative ones for the Al(iii)·Aβ complex. Interestingly, the same three residues (Glu3, Asp7, and Glu11) appear in the Al(iii) first coordination shell of these five structures so they are the most likely residues to be coordinated by Al(iii). Among them, Compl1^3,7,11^ is the most stable structure, with an interaction energy of –172.9 kcal mol^–1^. There, Glu3 and Asp7 interact monodentatedly with the cation, while Glu11 does bidentatedly. The peptide bond carbonyl oxygens of Glu3 and Glu11 also interact with Al(iii). The shortest distances with respect to Al(iii) correspond to O_δ2_@Asp7 (1.81 Å) and O_ε2_@Glu3 (1.86 Å), while the longest ones correspond to O@Glu11 (2.01 Å) and O_ε2_@Glu11 (2.01 Å). A slightly different arrangement is found in Compl2^3,4,7,11^, with a Δ*E*_aq_ value of –169.9 kcal mol^–1^. Even though the carboxylic groups of Asp7 and Glu11 interact with Al(iii), Glu3 only does so through its peptide carbonyl oxygen, while its carboxylic groups are facing the solvent. Two more carbonyl oxygen atoms (Phe4 and Glu11) complete the metal first coordination shell. In Compl3^3,7,9,11^, with a Δ*E*_aq_ value of –165.7 kcal mol^–1^, the same three residues (Glu3, Asp7, and Glu11) are coordinated to the cation, but in this case Glu3 binds monodentatedly, and Asp7 and Glu11 bidentatedly. The carbonyl oxygen of Gly9 also interacts with Al(iii).

The carboxylic groups of Glu3, Asp7, and Glu11 are also involved in the coordination of Al(iii) in Compl4^3,7,11,*W*^ and Compl5^3,7,11^, with Δ*E*_aq_ values of –165.1 and –158.6 kcal mol^–1^, respectively. The only difference between them, apart from the conformation of the Aβ peptide, is that the three carboxylic groups interact bidentatedly in Compl5^3,7,11^, while in Compl4^3,7,11,*W*^ Asp7 does monodentatedly and the free coordination place is occupied by a water molecule.

Compl6^1,3,7,*W*^ and Compl7^1,3,11^ are the first structures where the Asp1 amino acid interacts with Al(iii), along with Glu3 and Asp7 or Glu11. Thus, the coordination modes of Al(iii) in Compl6^1,3,7,*W*^ and Compl7^1,3,11^ resemble the ones found in Compl4^3,7,11,*W*^ and Compl5^3,7,11^, respectively. However, the substitution of Glu11 or Asp7 by Asp1 has increased their Δ*E*_aq_ values substantially (more than 25 kcal mol^–1^), suggesting that the inclusion of Asp1 in the metal first coordination shell destabilizes the complex.

In the remaining structures, which clearly show less favorable interaction energies (ΔΔ*E*_aq_ > 85 kcal mol^–1^), only two carboxylic groups are coordinated to Al(iii), while the coordination shells are fulfilled by two or three water molecules. The large gap in the Δ*E*_aq_ values of these two sets of structures agrees with the cluster model calculations, indicating that the reduction in the number of carboxylic groups interacting with Al(iii) has a drastic effect on the stability of the Al(iii)–Aβ complex, and that three carboxylic groups interacting with Al(iii) provides good stability to the complex.

It is noteworthy that in the most stable 3D models Al(iii) is coordinated into the 1–16 segment of the Aβ peptide, as Cu(ii) and Zn(ii) do. However, coordination features obtained for Al(iiii)–Aβ are significantly different to those determined for Cu(ii)–Aβ, which involves His6, His13 or His14, carbonyl oxygen, and the terminal NH2 in component I or His6, His13, His14 and CO(Ala2) for Component II.^7,30^ Thus, all of the carboxylate groups are exposed to the solvent in the Cu(ii)–Aβ complex, while in Al(iii)–Aβ three of them are interacting with the cation. This difference is expected to shape the aggregation pattern of each complex and accounts for the different morphologies shown by the aggregates grown in the presence of each of these cations.^19^

## Conclusions

By translating the pre-organization concept of host–guest interaction for the binding of metal to peptide, we here present relevant insights on the interaction of Al(iii) with Aβ and overcome the major limitation so far: the absence of any reliable template experimentally available of Al(iii) interacting with middle sized Aβ peptides. To reach final physically sound models, different computational approaches have been combined and this allows us to shed light on the preferential interaction mode between Al(iii) and an Aβ peptide and present, for the first time, 3D models of the complex. Remarkably, the results provide a detailed description at the atomistic scale of the Al(iii)–Aβ complex.

First, a large set of DFT cluster models were built to model alternative binding sites of Al(iii) and to infer its intrinsic coordination mode preferences. Al(iii) shows a marked preference for carboxylic groups and up to four of this type of group can interact with the cation in the most stable structures. The results also indicate that the metal coordination shell is completed by water molecules or amide bond carbonyl oxygens. All of these data provide useful information for proceeding to build reliable Al(iii)–Aβ complexes.

Next, a large number of structures of Aβ peptides in solution, solved by NMR, were used as tentative models for building the structures of the Al(iii)–Aβ complex. This large set of geometries provide enough sampling of different conformations of the Aβ peptide for finding pre-organized conformations of the peptide for adequately coordinating an Al(iii) cation. These structures were filtered using a 3D grid algorithm and then coordination rules were applied with the MOGA algorithm. The emerged structures were further refined by MD and QM/MM calculations in order to obtain reliable geometries of the complex and evaluate the interaction energies between Al(iii) and the Aβ peptide on them. This protocol gave rise to 13 model structures as the most suited to represent the Al(iii)–Aβ complex.

The analysis of these 13 structures indicates that the number of carboxylic groups determines the interaction energy between Al(iii) and Aβ, as those models with three carboxylic groups in the Al(iii) coordination shell are clearly the most stables ones. The study predicts that Glu3, Asp7, and Glu11 are the most suited amino acids to be coordinated by Al(iii), and that some peptide bond carbonyl oxygens may fill the metal coordination shell.

The study therefore proposes, for the first time, a 3D model for the Al(iii)–Aβ complex, and identifies the amino acids involved in the metal binding. The proposed coordination mode of Al(iii) to Aβ differs from the ones established for other cations. We believe that this is key information for investigating the biophysics of the Al(iii)–Aβ complex, which is fundamental to understand the biochemical role of Al(iii) in AD.

Additionally, the present work gives a proof-of-concept of an original multi-level computational pipeline. It overcomes the numerous barriers resulting from the very limited three dimensional information that was available at the beginning of the project. Using the hypothesis that close-to-native metallopeptide geometries are naturally explored by the unbounded form of the biopolymer, this strategy only relies on identifying those geometries inside the conformational ensemble provided by experimental structures of the Aβ peptide that are available in the Protein Data Bank, something reminiscent to strategies used for *de novo* enzyme design. Looking for such pre-organized forms of the coordinating molecule allows the issue of the lack of structural information on the Al(iii) metallopeptide to be solved and avoids the necessity of using a metallopeptide resulting from the fixation of chemically different metals as templates. Such a strategy has shown extremely encouraging results, and its applicability to other kinds of ensembles (*i.e.* like those obtained by long range molecular dynamics) is now under investigation. We expect that this could shed light on other molecular systems for which 3D models are difficult to generate *via* standard methodologies.
