# Supplementary material for: Elucidating the 3D structures of Al(iii)–Aβ complexes: a template free strategy based on the pre-organization hypothesis
Source: Chem Sci. 2017 May 9;8(7):5041–9. doi: 10.1039/c7sc01296a (PMC5613242; doi:10.1039/c7sc01296a)
Supplement: Supplementary file 1 [file SC-008-C7SC01296A-s001.pdf]

Electronic Supplementary Material (ESI) for Chemical Science.
This journal is © The Royal Society of Chemistry 2017
Please do not adjust margins

Journal Name

ARTICLE

ESI: Elucidating the 3D structures of Al(III)-Aβ complexes: a template free strategy based on the pre-organization hypothesis

Received 00th January 20xx,
Accepted 00th January 20xx

DOI: 10.1039/x0xx00000x
www.rsc.org/

Jon I. Mujika,[a]† Jaime Rodríguez-Guerra Pedregal,[b] Xabier Lopez,[a] Jesus M. Ugalde,[a] Luis Rodríguez-Santiago,[b] Mariona Sodupe,[b] and Jean-Didier Maréchal[b]†

1 Specific Computational Details

1.1 Cluster models

Geometry optimizations of all cluster models were carried out in solution with Integral Equation Formalism Polarizable Continuum Model (IEFPCM)[1] as implemented in Gaussian09.[2] The B3LYP functional[3–6] in conjunction with the 6-31+g(d) basis set was employed to optimize the geometries. Frequency calculations at the same level of theory were carried out to verify the nature of the stationary points encountered. All structures showed real frequencies for all the normal modes of vibration. The frequencies were then used to evaluate the zero-point vibrational energy (ZPVE) and the thermal (T = 298 K) vibrational corrections to the enthalpy in the harmonic oscillator approximation. The electronic energy was refined by single-point calculation in solution by a B3LYP/6-311++G(3df,2p) single-point calculation with IEFPCM at the B3LYP/6-31+G(d) geometries optimized in solution.
The stability of each complex was determined by calculating the reaction energy of the next reaction:

$$[Al.(\mathrm{H_2O})_6]^{3+}(\mathrm{aq,1M}) + \mathrm{m.}[\mathrm{Lig^{ni}}]^{\mathrm{p}}(\mathrm{aq,1M}) + \mathrm{n.}[\mathrm{Lig^{i}}]^{\mathrm{q}}(\mathrm{aq,1M}) \leftrightarrow [Al.Lig_{m+n}.(H_2O)_{6-m-n}]^{3+p+q}(aq,1M) + m.H_2O(aq,1M) + n.H_3O^+(aq,1M) \quad \mathrm{(S1)}$$

where *m* refers to the number of ligands which are not ionized (Lig^{ni}) upon their complexation to Al(III), while *n* refers to the number of ligands that are considered neutral in solution and unprotonated in the complex (Lig^{i}, see below); *p* and *q* refer to the total charge of Lig^{ni} and Lig^{i}, respectively. The enthalpy in solution is therefore calculated as:

$$\Delta H_{aq}^{298} = H_{aq}^{298}(Al.Lig_{m+n}(H_2O)_{6-m-n}) + m.H_{aq}^{298}(H_2O) + n.H_{aq}^{298}(H_3O) - H_{aq}^{298}(Al.(H_2O)_6) - m.H_{aq}^{298}(Lig^{ni}) - n.H_{aq}^{298}(Lig^{i}) + \Delta n.RTln(24.46) + m.RT(ln55.34) \quad (S2)$$

Since the solvation energies are determined using an ideal gas at 1 atm as the standard state, the penultimate term in Equation S2 corresponds to the volume change due to the transformation from 1 atm to 1 M, where Δn refers to the change in the number of species in the reaction. The last term accounts for the concentration of 55.34 M of water in liquid water.
The acidity of a molecule changes substantially upon its coordination to Al(III), what determines the protonation states of the titratable groups. For instance, a previous computational study reported that the pK_a of tyrosine drops from 10.1 in solution to 4.1 when interacting with Al(III).[7] Besides tyrosine, a deprotonation is also expected for the N-terminal when interacts with Al(III). Thus, these two groups are considered protonated in solution, but unprotonated when are coordinated to Al(III)(Lig^{i} in

[a.] *Kimika Fakultatea, Euskal Herriko Unibertsitatea (UPV/EHU) and Donostia International Physics Center (DIPC), 20080 Donostia, Euskadi (Spain)*
[b.] *Departament de Química, Universitat Autònoma de Barcelona, Bellaterra 08193, Spain*

†Corresponding authors: JIM (joni.mujika@ehu.eus) and JDM (JeanDidier.Marechal@uab.cat)

Please do not adjust margins

Please do not adjust margins

Equation S1). In addition, in order to account for the higher concentration of H_3O^+, the next correction was added:

$$\Delta G_{deprot} = 2.303RT(pK_a - pH) \qquad (S3)$$

where pK_a is the pK_a value of the deprotonated group (10.1 for tyrosine and 12.6 for N-terminal) and pH is the environment pH (7.4).

1.2 Structural Bioinformatics approach to generate first order Al(III)-Aβ models

On the basis of the most probable first coordination spheres of the Al(III) identified in the former step, a structural screening of experimental structure of amyloid was performed. To do so, a total of 13 PDB structures from the Protein Data Bank[8] were downloaded, representing up to 157 NMR models. Using a series of in-house python scripts, our methodology include two main parts.

First, a 3 dimensional grid encapsulating the entire peptide plus 5 Å were built for each structure. For each box, a grid with points spaced each 4 Å was generated. Any points at a minimum distance of 1 Å from any proteic atom were deleted. On the remaining points, and due to the high preference of Al(III) towards carboxylic groups inferred from cluster model calculations, the distances with respect to the six carboxylic groups (three aspartates and three glutamates) present in Aβ peptides ($A\beta_{42}$ peptide sequence shown in Figure 2) were calculated. Those points that show the C_α atoms of Asp or Glu closer than 7 Å (the average distance observed for C_α of coordinating Glutamate residues) were kept. Note that the C_α atoms of the Asp or Glu amino acids and not their side chains were taken as reference (see Figure S1). This criterion provides further flexibility to the side chain, and allows a specific search of the most stable rotamers. In 85 of the 157 structures downloaded from the PDB bank this criteria was satisfied. So, since in some structures more than one tentative location were found, finally a total of 194 positions were determined as suitable to place Al(III). Therefore, the amount of possibilities was still too large and a further discrimination was necessary, achieved by the search of rotamers and scoring of different Al(III)-Aβ structures with alternative coordination modes of Al(III).

Second, on the structures selected in the previous step, we applied a Multi-Objective Genetic Algorithm (MOGA). Our exploration was carried out using two genes: 1) the position of the metal ion and 2) the rotameric conformation of the side chain with respect to the metal environment. The evaluation (fitness) included the quality of the first coordination sphere of the metal in which all the coordinating atoms from the side chains Asp, Glu, His, and Arg and backbone atoms were considered. Coordination has been modeled through simple geometrical variables: distances, angles and dihedrals. These three variables (genes) were optimized so the values tend to: i) distance of 2.5 Å (to be not too much restrictive and allow possible further relaxation of the peptide in binding the metal), ii) angles Ligand-Metal-Ligand as close as possible to 90 degrees and iii) dihedral between the metal and the three atoms of the coordinating group to zero. The other evaluation function (objective) is the steric clashes which are asked to be minimized during the exploration.

1.3 Molecular Dynamics simulations

Molecular dynamics simulations were carried out by the CHARMM package (version c39b1).[9,10] The CHARMM36 all-atom force field was used to build the topology of the protein and the non-bonded parameters of Al(III) were taken from ref.,[11] as they provided satisfactory results in previous molecular dynamics simulations.[12] Once the system was built, the steepest descents (SD) method was employed to minimize the energy of the system and remove bad contacts. Periodic boundary conditions were applied in all directions using a rhombic dodecahedron cell, with a minimal distance between the protein and the wall of the cell set to 10 Å. Then, TIP3 water molecules[13] were added (the number of water molecules differs on each system due to different dimensions of the PBC cell), and the system neutralized with a NaCl concentration of 0.150 M. The energy of the entire system was further minimized with the SD algorithm.

The molecular dynamics simulations were carried out under canonical thermodynamic ensemble (NVT) using the Hoover thermostat. Long-range electrostatics were calculated using the smooth particle mesh Ewald (PME) method,[14,15] with a real space cutoff of 12 Å, a sixth-order cubic spline and a kappa value of 0.34 Å-1. A cut-off of 12 Å was defined for the Lennard-Jones non-bonded interaction, applying a switch function beyond 10 Å. The non-bond atom pair list were heuristically updated using a cutoff of 16 Å. All bond lengths involving hydrogens were constrained with SHAKE, allowing a time step of 1fs. The system was first heated from 110K to 310K by a 100 ps long simulation, and equilibrated by a 1ns long simulation at 310K. Finally, a simulation of 10 ns was carried out for each system.

1.4 QM/MM optimizations

Three structures were picked up from each molecular dynamics simulations (two intermediate structures and the last one). These geometries were optimized by QM/MM multiscale calculations using the pDynamo program,[16] and in particular the interface provided by this code with the Orca quantum package.[17] It must note that with this approach all non-bonded interactions are included, what implies a large computational time for calculating interactions far away from the metal binding site. Thus, the system was truncated at 18 Å from Al(III), and those atoms beyond 15 Å kept fixed. In this way, the interaction between Al(III) and Aβ peptide is properly described at a reasonable computational time.

The conjugate gradient algorithm was used to optimize the structures. For those covalent bonds between atoms located at the QM and MM regions, a hydrogen 'link atom' was added as the boundary of the QM region.[18] The QM part was treated at

 This journal is © The Royal Society of Chemistry 20xx

Please do not adjust margins

Please do not adjust margins

B3LYP/6-31+G(d) level of theory, where the MM charges were electronically embedded. The CHARMM36 force field was used for the MM part. For each QM/MM optimization, Al(III) and its first coordination shell were included in the QM part, what implies that different number of atoms described by the high level of theory may be included at each complex. A tolerance of 10^{-3} kcal/mol.Å in the energy gradient was defined for the optimizations.

1.5 Interaction energies

The structures optimized by QM/MM hybrid methods provide useful information about their geometry and about the specific coordination mode of Al(III) at each of these structures, but their energies cannot be compared directly because they include different number of atoms. Therefore, it cannot establish which coordination mode is energetically favorable. In order to address this, the interaction energy between Al(III) and Aβ peptide at each complex structure was determined by pure QM calculations. To do that, the $A\beta_{1-16}$ segment of the Aβ peptide was taken from each optimized structure, as in all systems the ligands coordinated to Al(III) are located in the 1-11 segment of the peptide. The new system also includes Al(III) and the water molecules forming the cation coordination shell. The interaction energy for each structure was evaluated according to the next equation:

$$[Al.(H_2O)_6]^{+3}+[A\beta_{1-16}]^{-1}\leftrightarrow[Al.A\beta_{1-16}.(H_2O)_{6-m}]^{+2}+m.H_2O \qquad (S4)$$

where $A\beta_{1-16}$ is the apoform of the 1-16 fragment of the Aβ peptide, $[Al.A\beta_{1-16}.(H_2O)_{6-m}]$ the complex formed by the Aβ peptide fragment, Al(III) and the water molecules placed in its solvation sphere, and *m* the number of water molecules displaced by the Aβ peptide from $Al.(H_2O)_6$. The structure of the apoform was taken from the complex, and therefore reaction S4 provides the interaction energy between Aβ peptide and Al(III) in the $[Al.A\beta_{1-16}.(H_2O)_{6-m}]$ complex structure.

The energy of each specie was determined in solution by a single-point calculations carried out with Turbomole[19] at the structures of $A\beta_{1-16}$ and $[Al.A\beta_{1-16}.(H_2O)_{6-m}]$ extracted from the QM/MM optimized geometries. In turn, the geometries of $Al.(H_2O)6$ and H_2O were fully optimized. Since the Resolution of Identity (RI) approximation was employed,[20,21] the non-hybrid PBE functional was chosen, combined with the def2-SV(P) basis set. The dispersion term was also included. The effect of the solvent was included by the COSMO continuum solvation model.[22,23]

Table S1: Set of 13 PDB files downloaded from PDB Bank. They contain a total of 157 structures of Aβ peptide that were used as templates to build the initial structures for the Al(III)-Aβ complex.

Aβ peptide length	Environment	PDB Code
1-28	In solution	1AMB, 1AMC, 1BJB, 1BJC
1-40	In solution	1AML
1-40	Water-micelle	1BA4
1-40	In solution, partially folded	2LFM
1-42	In solution	1IYT, 1ZOQ
1-40	In solution, but MET35SME	1BA6
1-29	Rat Aβ in solution, Zn	1NMJ
1-40	Interacting with a molecule	2M9R, 2M9S

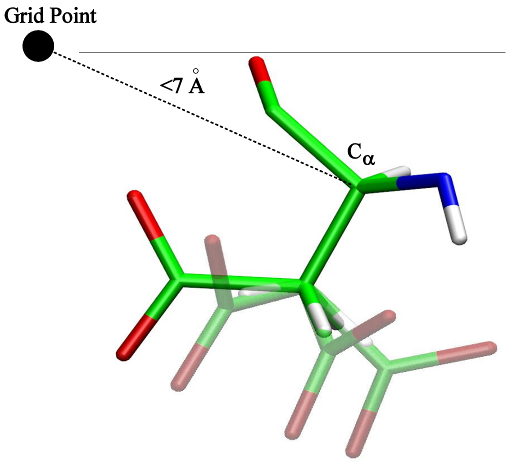

Figure S1: An Al(III) atom will be placed in a given grid point when at least the C_α carbon of three Asp or Glu amino acids are closer than 7 Å. This criterion provides a larger flexibility to the carboxylic side chains to be accommodated in the metal coordination shell.

Please do not adjust margins

Please do not adjust margins

Table S2 Distances between Al(III) and the oxygen atoms located at its first coordination shell computed on the most stable structures among the three geometries optimized for each of the 13 Al(III)-Aβ complexes. The interaction energies computed on the 13 structures (ΔEaq, in kcal/mol) are given along with the average energy values (<ΔEaq>, in kcal/mol) computed on the three structures. The superscripts refer to the ligands interacting with Al(III) at each complex.

Complex	Asp1		Glu3		Asp7		Glu11		Carbonyl	H_2O	ΔE_{aq}	**$\langle\Delta E_{aq}\rangle$**
	$O_{\delta1}$	$O_{\delta2}$	$O_{\varepsilon1}$	$O_{\varepsilon2}$	$O_{\delta1}$	$O_{\delta2}$	$O_{\varepsilon1}$	$O_{\varepsilon2}$	O	O_W		
Complex1^{3,7,11}	–	–	4.03	1.86	3.80	1.81	1.92	2.01	1.93(Glu3)<br>2.01(Glu11)	–	-178.4	**-172.9**
Complex2^{3,4,7,11}	–	–	–	–	1.94	1.94	1.81	3.93	1.87(Gly3)<br>1.90(Phe4)<br>1.89(Gly11)	–	-179.1	**-169.9**
Complex3^{3,7,9,11}	–	–	1.86	3.48	1.99	1.93	2.06	1.94	1.86(Glu9)	–	-170.2	**-165.7**
Complex4^{3,7,11,W}	–	–	2.02	1.92	3.28	1.84	1.93	2.01	–	1.91	-174.5	**-165.1**
Complex5^{3,7,11}	–	–	1.95	1.94	1.94	1.93	1.94	1.93	–	–	-165.6	**-158.6**
Complex6^{1,3,7,W}	1.98	1.98	3.40	1.83	2.01	1.92	–	–	–	1.89	-147.8	**-140.3**
Complex7^{1,3,11}	1.99	1.93	1.92	1.95	–	–	1.92	1.95	–	–	-132.9	**-130.5**
Complex8^{7,11,W}	–	–	–	–	1.94	1.99	1.95	1.92	–	1.90	-91.1	**-86.9**
Complex9^{7,11,2W}	–	–	–	–	1.93	1.95	1.98	1.96	–	1.90,1.91	-95.0	**-78.2**
Complex10^{3,11,3W}	–	–	1.89	3.64	–	–	1.94	2.00	–	1.92–1.94	-78.8	**-64.2**
Complex11^{3,7,2W}	–	–	1.93	1.96	2.02	1.90	–	–	–	1.89,1.89	-61.5	**-57.0**
Complex12^{1,3,2W}	2.00	1.96	1.97	1.93	–	–	–	–	–	1.89,1.90	-44.6	**-41.6**
Complex13^{1,7,3W}	3.99	1.81	–	–	1.94	2.02	–	–	–	1.91–1.94	-47.3	**-40.1**

Table S3 Distances between Al(III) and the oxygen atoms located at its first coordination shell computed on the second most stable structures among the three geometries optimized for each of the 13 Al(III)-Aβ complexes. The interaction energies computed on the 13 structures (ΔEaq, in kcal/mol) are given along with the average energy values (<ΔEaq>, in kcal/mol) computed on the three structures. The superscripts refer to the ligands interacting with Al(III) at each complex.

Complex	Asp1		Glu3		Asp7		Glu11		Carbonyl	H_2O	ΔE_{aq}	**$\langle\Delta E_{aq}\rangle$**
	$O_{\delta1}$	$O_{\delta2}$	$O_{\varepsilon1}$	$O_{\varepsilon2}$	$O_{\delta1}$	$O_{\delta2}$	$O_{\varepsilon1}$	$O_{\varepsilon2}$	O	O_W		
Complex1^{3,7,11}	–	–	3.99	1.84	3.81	1.81	1.93	2.05	1.93(Glu3)<br>2.08(Glu11)	–	-175.4	**-172.9**
Complex2^{3,4,7,11}	–	–	–	–	1.97	1.92	1.81	3.95	1.86(Gly3)<br>1.91(Phe4)<br>1.93(Gly11)	–	-168.3	**-169.9**
Complex3^{3,7,9,11}	–	–	1.85	3.68	1.95	1.96	2.02	1.93	1.89(Glu9)	–	-168.2	**-165.7**
Complex4^{3,7,11,W}	–	–	2.00	1.92	3.80	1.81	1.98	1.96	–	1.90	-160.9	**-165.1**
Complex5^{3,7,11}	–	–	1.93	1.97	1.97	1.91	1.94	1.93	–	–	-155.6	**-158.6**
Complex6^{1,3,7,W}	1.95	1.99	3.78	1.80	2.14	1.89	–	–	–	1.87	-138.0	**-140.3**
Complex7^{1,3,11}	1.97	1.95	1.93	1.91	–	–	1.92	1.93	–	–	-131.8	**-130.5**
Complex8^{7,11,W}	–	–	–	–	1.90	1.99	1.92	1.96	–	1.90	-91.1	**-86.9**
Complex9^{7,11,2W}	–	–	–	–	1.93	1.98	1.95	1.94	–	1.88,1.92	-70.5	**-78.2**
Complex10^{3,11,3W}	–	–	1.84	3.45	–	–	1.97	1.95	–	1.89–1.97	-58.4	**-64.2**
Complex11^{3,7,2W}	–	–	1.95	1.94	2.00	1.93	–	–	–	1.89,1.89	-56.5	**-57.0**
Complex12^{1,3,2W}	1.96	1.96	1.96	1.92	–	–	–	–	–	1.87,1.91	-40.5	**-41.6**
Complex13^{1,7,3W}	3.54	1.80	–	–	1.97	1.94	–	–	–	1.92–1.95	-38.2	**-40.1**

This journal is © The Royal Society of Chemistry 20xx

Please do not adjust margins

Table S4 Distances between Al(III) and the oxygen atoms located at its first coordination shell computed on the third most stable structures among the three geometries optimized for each of the 13 Al(III)-Aβ complexes. The interaction energies computed on the 13 structures (ΔEaq, in kcal/mol) are given along with the average energy values (<ΔEaq>, in kcal/mol) computed on the three structures. The superscripts refer to the ligands interacting with Al(III) at each complex.

Complex	Asp1		Glu3		Asp7		Glu11		Carbonyl	H_2O	ΔE_{aq}	**<ΔE_{aq}>**
	$O_{\delta1}$	$O_{\delta2}$	$O_{\varepsilon1}$	$O_{\varepsilon2}$	$O_{\delta1}$	$O_{\delta2}$	$O_{\varepsilon1}$	$O_{\varepsilon2}$	O	O_W		
Complex1[3,7,11]	–	–	1.86	3.79	3.70	1.81	1.92	2.01	1.94(Glu3)<br>1.96(Glu11	–	-164.9	**-172.9**
Complex2[3,4,7,11]	–	–	–	–	1.95	1.95	1.80	3.87	1.85(Gly3)<br>1.90(Phe4)<br>1.90(Gly11)	–	-162.2	**-169.9**
Complex3[3,7,9,11]	–	–	1.86	3.82	1.99	1.94	2.11	1.89	1.87(Glu9)	–	-158.6	**-165.7**
Complex4[3,7,11,W]	–	–	1.99	1.93	3.54	1.83	1.91	2.02	–	1.96	-159.9	**-165.1**
Complex5[3,7,11]	–	–	2.00	1.92	1.97	1.92	1.97	1.90	–	–	-154.6	**-158.6**
Complex6[1,3,7,W]	1.94	2.02	3.70	1.85	1.99	1.90	–	–	–	1.90	-135.2	**-140.3**
Complex7[1,3,11]	1.96	1.97	1.93	1.91	–	–	1.96	1.92	–	–	-126.8	**-130.5**
Complex8[7,11,W]	–	–	3.99	1.84	3.81	1.81	1.93	2.046	–	1.90,193	-78.5	**-86.9**
Complex9[7,11,2W]	–	–	–	–	1.93	1.94	1.98	1.94	–	1.90,1.94	-69.1	**-78.2**
Complex10[3,11,3W]	–	–	1.82	3.88	–	–	1.93	1.97	–	1.94–1.97	-55.5	**-64.2**
Complex11[3,7,2W]	–	–	1.92	1.96	2.03	1.89	–	–	–	1.89,1.89	-53.0	**-57.0**
Complex12[1,3,2W]	1.97	1.96	1.97	1.92	–	–	–	–	–	1.89,1.90	-39.6	**-41.6**
Complex13[1,7,3W]	3.64	1.82	–	–	1.97	1.97	–	–	–	1.89–196	-34.8	**-40.1**

References

1 J. Tomasi, B. Mennucci and R. Cammi, *Chem Rev*, 2005, 2999–3093.

2 M. J. Frisch, G. W. Trucks, H. B. Schlegel, G. E. Scuseria, M. A. Robb, J. R. Cheeseman, G. Scalmani, V. Barone, B. Mennucci, G. A. Petersson, H. Nakatsuji, M. Caricato, X. Li, H. P. Hratchian, A. F. Izmaylov, J. Bloino, G. Zheng, J. L. Sonnenberg, M. Hada, M. Ehara, K. Toyota, R. Fukuda, J. Hasegawa, M. Ishida, T. Nakajima, Y. Honda, O. Kitao, H. Nakai, T. Vreven, J. A. Montgomery Jr., J. E. Peralta, F. Ogliaro, M. Bearpark, J. J. Heyd, E. Brothers, K. N. Kudin, V. N. Staroverov, R. Kobayashi, J. Normand, K. Raghavachari, A. Rendell, J. C. Burant, S. S. Iyengar, J. Tomasi, M. Cossi, N. Rega, J. M. Millam, M. Klene, J. E. Knox, J. B. Cross, V. Bakken, C. Adamo, J. Jaramillo, R. Gomperts, R. E. Stratmann, O. Yazyev, A. J. Austin, R. Cammi, C. Pomelli, J. W. Ochterski, R. L. Martin, K. Morokuma, V. G. Zakrzewski, G. A. Voth, P. Salvador, J. J. Dannenberg, S. Dapprich, A. D. Daniels, Ö. Farkas, J. B. Foresman, J. V. Ortiz, J. Cioslowski and D. J. Fox, *Gaussian 09 Revision A.1*, .

3 A. D. Becke, *Phys Rev A*, 1988, **38**, 3098–3100.

4 A. D. Becke, *J Chem Phys*, 1993, **98**, 5648–5652.

5 C. Lee, W. Yang and R. G. Parr, *Phys Rev B*, 1988, **37**, 785–789.

6 S. H. Vosko, L. Wilk and M. Nusair, *Can J Phys*, 1980, **58**, 1200–1211.

7 J. I. Mujika, J. M. Ugalde and X. Lopez, *Theor Chem Acc*, 2011, **128**, 477–484.

8 H. M. Berman, J. Westbrook, Z. Feng, G. Gilliland, T. N. Bhat, H. Weissig, I. N. Shindyalov and P. E. Bourne, *Nucleic Acids Res.*, 2000, **28**, 235–242.

9 B. R. Brooks, C. L. Brooks III, A. D. Mackerell Jr., L. Nilsson, R. J. Petrella, B. Roux, Y. Won, G. Archontis, C. Bartels, S. Boresch, A. Caflisch, L. Caves, Q. Cui, A. R. Dinner, M. Feig, S. Fischer, J. Gao, M. Hodoscek, W. Im, K. Kuczera, T. Lazaridis, J. Ma, V. Ovchinnikov, E. Paci, R. W. Pastor, C. B. Post, J. Z. Pu, M. Schaefer, B. Tidor, R. M. Venable, H. L. Woodcock, X. Wu, W. Yang, D. M. York and M. Karplus, *J Comput Chem*, 2009, **30**, 1545–1614.

10 B. R. Brooks, R. E. Bruccoleri, D. J. Olafson, D. J. States, S. Swaminathan and M. Karplus, *J Comput Chem*, 1983, **4**, 187–217.

11 T. M. C. Faro, G. P. Thim and M. S. Skaf, *J Chem Phys*, 2010, **132**, 114509.

12 J. I. Mujika, B. Escribano, E. Akhmatskaya, J. M. Ugalde and X. Lopez, *Biochemistry (Mosc.)*, 2012, **51**, 7017–27.

13 W. L. Jorgensen, J. Chandrasekhar, J. D. Madura, R. W. Impey and M. L. Klein, *J Chem Phys*, 1983, **79**, 926–935.

14 T. Darden, D. York and L. Pedersen, *J Chem Phys*, 1993, **98**, 10089–10092.

15 U. Essmann, L. Perera, M. L. Berkowitz, T. Darden, H. Lee and L. G. Pedersen, *J Chem Phys*, 1995, **103**, 8577–8592.

16 M. J. Field, *J Chem Theory Comput*, 2008, **4**, 1156–1161.

17 F. Neese, *Wiley Interdiscip. Rev. Comput. Mol. Sci.*, 2012, **2**, 73–78.

18 Frontier Bonds in QM/MM Methods: A Comparison of Different Approaches - The Journal of Physical Chemistry A (ACS Publications), http://pubs.acs.org/doi/abs/10.1021/jp9924124?journalCode=jpcafh&quickLinkVolume=104&quickLinkPage=1720&selectedTab=citation&volume=104, (accessed April 26, 2017).

19 *Turbomole V6.6 2014, a development of University of Karlsruhe and Forschungszentrum Karlsruhe GmbH, 1989-2007, Turbomole GmbH, since 2007; available from \tt http://www.turbomole.com.*, .

20 K. Eichkorn, O. Treutler, H. Ohm, M. Haser and R. Ahlrichs, *Chem Phys Lett.*, 1995, **242**, 652.

21 K. Eichkorn, F. Weigend, O. Treutler and R. and Ahlrichs, *Theor Chem Acc*, 1997, **97**, 119.

22 A. Klamt and G. Schueuermann, *J Chem Soc Perkin Trans 2*, 1993, 799–805.

23 S. Sinnecker, A. Rajendran, A. Klamt, M. Diedenhofen and F. Neese, *J Phys Chem A*, 2006, **110**, 2235–2245.

This journal is © The Royal Society of Chemistry 20xx

Please do not adjust margins

Please do not adjust margins
